# Urban and rural variations in morbidity and mortality in Northern Ireland

**DOI:** 10.1186/1471-2458-7-123

**Published:** 2007-06-26

**Authors:** Gareth O'Reilly, Dermot O' Reilly, Michael Rosato, Sheelah Connolly

**Affiliations:** 1 The Department of Epidemiology & Public Health, The Queen's University of Belfast, Belfast, Northern Ireland

## Abstract

**Background:**

From a public health perspective and for the appropriate allocation of resources it is important to understand the differences in health between areas. This paper examines the variations in morbidity and mortality between urban and rural areas.

**Methods:**

This is a cohort study looking at morbidity levels of the population of Northern Ireland at the time of the 2001 census, and subsequent mortality over the following four years. Individual characteristics including demographic and socio-economic factors were as recorded on census forms. The urban-rural nature of residence was based on census areas (average population c1900) classified into eight settlement bands, ranging from cities to rural settlements with populations of less than 1000.

**Results:**

The study shows that neither tenure nor car availability are unbiased measures of deprivation in the urban-rural context. There is no indication that social class is biased. There was an increasing gradient of poorer health from rural to urban areas, where mortality rates were about 22% (95% Confidence Intervals 19%–25%) higher than the most rural areas. Differences in death rates between rural and city areas were evident for most of the major causes of death but were greatest for respiratory disease and lung cancer. Conversely, death rates in the most rural areas were higher in children and adults aged less than 20.

**Conclusion:**

Urban areas appear less healthy than the more rural areas and the association with respiratory disease and lung cancer suggests that pollution may be a factor. Rural areas however, have higher death rates amongst younger people, something which requires further research. There is also a need for additional indicators of deprivation that have equal meaning in urban and rural areas.

## Background

One of the most important tasks that government can do to reduce health inequalities is to ensure that the appropriate amount and type of resources are directed to those most in need. Whilst this is normally interpreted in the form of deprivation the principle of fair apportionment is as applicable to urban and rural areas, which are arguably more identifiable for targeting purposes. It is therefore important to be able to characterise and quantify any differences in health and disease patterns between these areas.

There are good reasons to believe that the pattern of health varies between urban and rural areas, though there is a dearth of research evidence specific to Northern Ireland. Urban areas are associated with high levels of physical and social factors that are potentially detrimental to maintaining good health such as air pollution [1], stress [2] and fear of crime [3]. There may be a concentration of problems such as drug and alcohol abuse, which is contributed to by the downward social mobility associated with migration towards larger conurbations [4-6]. Social networks, which are useful in maintaining and protecting health, may be more fragmented, leading to increased mental ill-health. There is a widespread belief that rural areas do not suffer from the social problems seen in the city and are relatively healthy [7], though it has also been argued that socio-economic problems are more visible in urban areas, as rural communities are more diverse, with some of the poorest in the land interspersed amongst some very wealthy landowners, commuters and professionals [8]. Furthermore, access to services, including health services, is notably worse in rural areas [3].

The research question addressed in this paper is whether urban and rural areas have different patterns of health, even after controlling for differences in the types of people who live in these areas? Such a finding would suggest that the characteristics of the area of residence exert an additional, independent influence on health. This is known as the context-content debate [9].

## Methods

The data used in this project has been derived from a record linkage exercise undertaken by the Northern Ireland Statistics and Research Agency (NISRA) in which the enumerated 2001 Census population was linked with subsequent deaths registered over the following four years. These data allow analysis of both differences at the time of the census and a four-year longitudinal study of the mortality patterns from the time of the census. These anonymised data were held in a safe setting by NISRA and made available to the researchers for the purposes of this study. Ethical permission was not required for the use of these data.

All characteristics of the study population were as described on the census record. As well as the usual demographic variables of age, sex and marital status, religious denomination was included and dichotomised as Roman Catholic; non-Roman Catholic, of whom the overwhelming majority were Protestant. Previous studies of the Northern Ireland population have shown differences between these two groupings [10,11]. When comparing differences in health between areas it is obviously important to control for differences in demographic characteristics such as age and sex. It is also important to adjust for differences in socio-economic status, though this is difficult as it is possible that some indicators (such as car ownership and tenure) may not reflect an equivalent level of disadvantage in urban and rural areas. Three measures of disadvantage were considered for inclusion in the analysis: tenure (categorised as owner, private renting, social renting), car availability (categorised as two or more cars, one car, or no car), and social class using the National Statistics-Socioeconomic Classification [12] (NS-SEC, divided into eight categories (see table 1)). Initial analysis confirmed that car access and tenure could not be interpreted in the same way across the urban-rural spectrum, leaving only social class as the primary indicator of disadvantage. In the 2001 UK Census this was coded for people up to the age of 74.

**Table 1:** Distribution of demographic, social and socio-demographic characteristics of settlements across Northern Ireland according to census in 2001 (data presented as percentages for enumerated individuals only; all ages)

	Belfast Urban Area	Derry Urban Area	Large Towns	Medium Towns	Small Towns	Intermediate Settlement	Village	Rural^$^	All N. Ireland
**Population**									
Total (no.)	545,955	84,739	214,698	95,992	96,764	64,869	65,716	434,491	1,603,224
less than 25	34.5	41.6	35.2	37.5	34.6	36.1	35.9	36.4	35.8
65 or over	15.3	9.9	12.8	12.0	14.6	11.9	12.6	13.0	13.6
**Marital status**									
Married	37.8	33.1	39.5	37.9	40.9	42.2	41.1	43.9	39.9
Never married	48.1	54.8	47.1	49.9	46.1	46.7	47.4	47.9	48.2
Wid/sep/div	14.1	12.1	13.4	12.1	13.0	11.1	11.6	8.2	11.9
**Denomination**									
RC	32.1	75.2	39.1	59.2	36.8	39.6	42.0	45.5	41.6
**Tenure**									
Owner	74.2	67.0	75.9	75.0	78.0	83.0	77.3	90.4	79.3
Private Rental	5.9	4.7	5.2	5.7	7.1	4.0	5.1	4.0	5.2
Social Renting	19.8	28.3	18.9	19.4	14.8	12.9	17.6	5.5	15.5
**Car ownership**									
Two plus	31.6	24.3	30.6	34.2	38.8	43.6	38.4	58.5	39.7
One only	43.4	48.4	47.5	46.1	44.7	44.0	45.8	35.3	42.4
No access	25.0	27.3	21.9	19.7	16.5	12.5	15.8	6.2	17.9
**Social Class (NS-SEC)**									
Professional	31.6	26.6	25.8	26.3	29.0	31.8	24.1	24.4	27.9
Intermediate	9.8	7.1	8.8	8.6	8.8	9.1	7.9	5.8	8.2
Small employers	7.2	7.9	8.9	11.1	12.8	12.1	13.4	27.6	14.0
Lower Supervisory	10.2	10.9	12.1	9.9	10.1	10.3	11.1	8.3	10.0
Routine	28.4	33.9	34.2	33.0	27.8	27.8	32.4	23.9	28.6
Not working	5.3	9.2	5.0	6.0	4.4	3.9	5.5	4.4	5.2
Students	1.1	0.8	0.3	0.3	1.3	0.2	0.2	0.2	0.6
Unclassified	6.4	3.6	4.9	4.7	5.8	4.8	5.3	5.3	5.5

**$ **Rural is short hand for 'small village, hamlet and open countryside'

Source: Northern Ireland Longitudinal Study (Mortality Study)

There is no universally agreed definition of what constitutes an 'urban' or 'rural' area ^13^. Population density is often used, as the data are readily available, though this could lead to the misclassification of sparsely populated inner city areas. Others have used indicators which incorporated access to services and the proportion of population employed in agriculture [14]. In this study the recently constructed classification of settlements was used to define urban and rural areas. This was based on the study of large scale maps and ariel photographs, and is similar to that used throughout Britain [15]. In Northern Ireland eight settlement bands are recognised ranging from Band A (the Belfast Metropolitan Urban Area) to Band H (representing open countryside and settlements of less than 1000 people). The full classification was used for the initial descriptive analysis but was collapsed into more manageable aggregates for the analysis of health differences. The geographical unit used to designate area characteristics was the 'super output area'. This is derived from the census output geography and produces units with an average population size of 1894 persons (range 1300–2956).

The two census-based health questions (limiting long-term illness (LLTI) and general health in the preceding year (GH) [16]) were used to indicate levels of self-reported health status at baseline. LLTI, which measures predominantly physical aspects of health [17], provided a dichotomy of yes/no responses; while GH, which also encompasses mental aspects of health [18], offered three responses: 'good', 'fairly good' and 'not good'. Multivariate logistic regression was used to assess urban-rural differences in morbidity while controlling for other potentially important demographic and social factors. Deaths during the four years following the census were used to characterise the mortality differences across the urban-rural spectrum. Graphs of the age-specific mortality rates were used to examine for variation in death rates across the age-range and Cox's proportional hazards modelling was used to study the relationship between area characteristics and subsequent mortality. This controls for potential confounders, including the initial health status of the cohort members. Urban-rural differences in cause-specific mortality was explored by dividing deaths into seven commonly used broad classification groups; all circulatory disease (I00-I99); ischaemic heart disease (I20-I25); cerebrovascular disease (I60-69); all respiratory disease (J00-J99); all malignat cancer (C00-C97); lung cancer (C33-C34); breast cancer (C50); external causes (V01-Y98); accidents (V01-V99 and W00-X59); definite and probable suicide (X60-X84, Y10-Y34, Y870); and other causes. Most analyses were restricted to individuals enumerated in the census for whom there was complete information on health status and cause of death. In general the analysis of morbidity and mortality was limited to people aged 25–74 to maintain the relevance of the socio-economic variables; the exception to this was the basic descriptive statistics and the age-specific mortality rates, which were not adjusted for potential confounders and therefore could utilise the full age range.

## Results

Table 1 shows the characteristics of the population living in the different types of settlement in Northern Ireland at the time of the 2001 census. The Belfast and Derry urban areas combined account for almost 40% of the population, 25.4% live in towns of various sizes and 27.1% live in rural areas. There are no discernable urban-rural patterns in age distribution, though it is obviously affected by denomination, as the areas with a higher proportion of Catholics tend to have younger populations. More rural areas tend to have greater proportions of married people and lower proportions of people who are widowed, separated or divorced.

The patterns of socio-economic disadvantage vary according to the indicator of disadvantage selected. The prevalence of owner-occupation increases the more rural the area of residence and the proportion of social renting declines. Similarly, the proportion of people with access to two or more cars is higher in rural areas while the proportion without access to a car is much higher in urban areas. On the other hand, urban differences in social class are more difficult to discern. The proportion of professional and senior managers is slightly higher in urban areas but so also is the proportion of people employed in routine occupations. The major difference however, is in the proportions of people who were small employers or self-employed, which is almost four times as common in rural as in urban areas. Therefore, variations in both tenure and car ownership suggest higher levels of disadvantage in urban areas, a pattern not consistent with the social class indicators. This suggests that the value of the indicators may be context specific. If car ownership and tenure are fair indicators of socio-economic status across the urban-rural divide then we would expect people of the same social class to display similar levels of car and house ownership across the urban-rural spectrum. This, however, is not what is seen. Only 9% of rural dwelling people in social class groups NS-SEC 4–6 (representing the manual and more routine occupations) do not own a car and 11% are in social renting, compared to 37% and 33% respectively of their city dwelling peers. For this reason we have not used car availability or tenure to indicate socio-economic disadvantage in urban and rural areas in the rest of this paper.

Table 2 shows the distribution of morbidity indicators across the urban-rural categories. The pattern is the same whether morbidity is assessed using LLTI or GH, with a gradient of increasing wellbeing from city to rural areas. Those in the more rural areas are about 20% less likely to report a limiting long-term illness or to say that their general health over the last year was fair or not good. This pattern is little affected by adjustment for differences in demographic composition or socio-economic factors.

**Table 2:** Relationship between urban-rural character of area and the likelihood of (a) limiting illness or (b) poor general health, in people aged 25–74 years. Data represents odds ratios (95% confidence intervals) from logistic regression models.

(a) Odds ratio of reporting limiting long-term illness *			
	**Unadjusted**	**Adjusted for age & sex**	**Fully Adjusted^$^**

**City**	1.00	1.00	1.00
**Intermediate**	0.93 (0.92, 0.94)	0.95 (0.94, 0.96)	0.93 (0.92, 0.94)
**Rural**	0.81 (0.79, 0.83)	0.78 (0.77, 0.79)	0.81 (0.80, 0.82)

(b) Odds ratio of reporting that general health in the last year was 'fair' or 'not good' *			

	**Unadjusted**	**Adjusted for age & sex**	**Fully Adjusted^$^**

**City**	1.00	1.00	1.00
**Intermediate**	0.93 (0.92, 0.94)	0.96 (0.95, 0.97)	0.94 (0.93, 0.95)
**Rural**	0.80 (0.79, 0.81)	0.78 (0.77, 0.79)	0.81 (0.80, 0.82)

Source: Northern Ireland Longitudinal Study (Mortality Study)

**$ **= fully adjusted for age, sex, marital status, denomination and social class (NS-SEC)

* = Statistical significance: All significant at the P < 0.001 level

**Figure 1:**
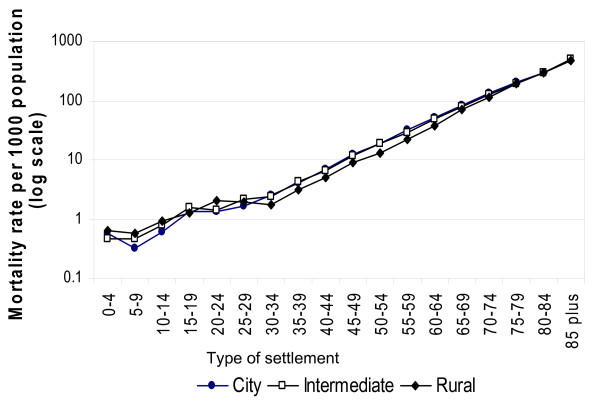
Age-specific mortality rates according to urban-rural character of area of residence, based on four years deaths following the 2001 census.

Figure 1 shows the age-specific mortality rates for each of the different settlement groups. These are shown on a logarithmic scale to offset the steep rise in mortality with age. Three distinct patterns are clear. For most of adulthood, the mortality experience of the most rural areas is significantly better than for the other areas, and there is little difference between the cities and the intermediate areas. The mortality experiences of these areas converge at older ages so that the context appears almost irrelevant for those aged 80 and over. For people aged less than 15 however, the pattern is reversed and death rates are lower for children in the city and highest in the most rural areas; mortality rates are also highest in rural areas for adults aged 20–24 years.

There were 20,699 deaths to Northern Ireland residents aged 25–74 years in the four years following the census. Table 3 shows the relative hazards of dying according to settlement type. These data, unlike the age-specific rates, have been adjusted for differences in the age, sex, marital status, denomination, and social class of the individuals. Overall, the risk of dying during the follow-up period was highest in the more urbanized areas. Residents of the rural areas had a risk of mortality 22% lower than those in the city while people in the intermediate areas had a 6% lower risk. Rural dwellers had a lower mortality from most of the major causes of death with the exception of breast cancer, though this was not statistically significant. Deaths due to external causes, including accidents, tended to be higher in more rural areas, though because the numbers of deaths were small these differences were not significant at the 5% level of significance. Differences between rural and city areas were particularly marked for respiratory disease and lung cancer.

**Table 3:** Variations in cause specific mortality in Northern Ireland for 'people aged 25–74 years, by area of residence. Data represent hazards ratios (95% confidence intervals) from Cox's proportional hazards modeling, fully adjusted for age, sex, marital status, denomination and social class.

**Cause of Death **(no. of deaths)	**City**	**Intermediate**	**Rural**
**All causes **(20,679)	1.00	0.94 *** (0.91, 0.97)	0.78 *** (0.75, 0.81)
**Circulatory disease **(6,711)	1.00	0.99 (0.94, 1.05)	0.84 *** (0.79, 0.90)
**Ischaemic heart disease **(4,086)	1.00	1.03 (0.96, 1.11)	0.88 *** (0.81, 0.96)
**Stroke **(1,362)	1.00	0.85 * (0.75, 0.97)	0.73 *** (0.64, 0.84)
**Respiratory disease **(2,015)	1.00	0.83 *** (0.75, 0.91)	0.62 *** (0.55, 0.7)
**All Malignancies **(7,867)	1.00	0.92 *** (0.87, 0.97)	0.79 *** (0.74, 0.84)
**Lung cancer **(1,890)	1.00	0.78 *** (0.7, 0.87)	0.57 *** (0.5, 0.65)
**Breast **(694)	1.00	1.03 (0.86, 1.22)	0.93 (0.77, 1.13)
**All external causes **(852)	1.00	1.14 (0.97, 1.33)	1.10 (0.92, 1.31)
**Accidents **(470)	1.00	1.11 (0.89, 1.37)	1.13 (0.90, 1.43)
**Suicides **(339)	1.00	1.18 (0.92, 1.51)	1.05 (0.79, 1.39)
**Other Causes **(3,234)	1.00	0.90 ** (0.83, 0.97)	0.66 *** (0.60, 0.73)

Statistical significance: *** P < 0.001; ** P < 0.01; * P < 0.05

Source: Northern Ireland Longitudinal Study (Mortality Study)

## Discussion

This study shows that, at the start of the 21st Century, 40% of the Northern Irish population lives in or around the cities of Belfast and Derry, followed in terms of size by the 27% who live in the most rural areas. Although there was some modest socio-demographic variation across the urban-rural spectrum, the greatest disparities were by tenure and car availability and the analysis suggests that their relationship to disadvantage varies across the urban-rural spectrum. The relatively short distances and ready availability of public transport makes car ownership less of a necessity in the city, while the higher house prices and greater availability of public sector housing increases the likelihood of renting. We conclude that only social class can act as a fair indicator of disadvantage across the urban-rural spectrum.

To our knowledge this is the first study using individual-level data to examine the demographic, socio-economic and health variations of Northern Ireland the population in terms of the urban-rural spectrum. The results show a trend, with more rural areas generally being more healthy. This was true both in terms of morbidity, as assessed by census-based measures of LLTI and GH, and all-cause mortality. The only exception was the higher mortality rates in children and adolescents in the more rural areas. Mortality in urban areas was higher from most of the major causes of deaths such as circulatory and respiratory disease, and cancer. The greatest urban-rural disparities were due to respiratory disease and lung cancer, suggesting that there are additional factors deleterious to respiratory health in large conurbations. As far back as the 17^th ^Century John Graunt noted the higher mortality in London than the countryside and attributed this to urban environmental pollution (primarily the burning of sulphurous coals) [19], and this has been confirmed by more recent studies relating air-quality and the health of city-dwellers [20]. A second possibility is that there has been insufficient adjustment in the analysis for material disadvantage, as both respiratory disease and lung cancer exhibit marked socio-economic gradients [21], primarily due to the association between socio-economic status and smoking. This cannot be examined directly using the current data but the study shows the need for an indicator of disadvantage that can be used 'fairly' over both urban and rural areas. Finally, it is possible that the differences may have arisen due to the selective movement of healthy and less health people between areas. Again this is not a new idea, for Welton [22] in 1872 (and later Hill [23], in 1925) suggested that the pattern of health could have arisen because '*it is the stronger [healthier] element that tends to migrate, while the weaker element tends to remain at home'*. We are currently exploring the contribution of selective migration to the spatial distribution of health across urban and rural areas.

The generally higher urban mortality rates should not detract from the recognition that each type of area is likely to have its own particular problems. The higher mortality amongst children and younger people in the rural areas is of particular concern and, although further analysis is necessary, it is probable that deaths due to external causes such as accidents are disproportionately higher in rural areas. The recent report on farmers and farming families in Northern Ireland [24] has highlighted the frequency of accidents and the high levels of stress anxiety and depression amongst this community. This suggests that health care planning, whilst acknowledging the generally poorer health status and higher mortality levels in cities, should be sufficiently sensitive to the differing health needs that the communities in urban and rural areas present.

## Conclusion

It is evidence that urban and rural areas present different socio-demographic characteristics and health profiles. It is not however possible to conclude that cities are inherently detrimental to health as their higher morbidity and mortality levels could be due to higher levels of deprivation which cannot be adequately adjusted for in the current datasets.

## Competing interests

The author(s) declare that they have no competing interests.

## Authors' contributions

DOR and SC devised the study; DOR, MR and GOR undertook the analysis; GOR wrote the first draft; all authors read and approved the final draft. DOR is the guarantor.

## Pre-publication history

The pre-publication history for this paper can be accessed here:


